# Broad-spectrum anti-HIV activity and high drug resistance barrier of lipopeptide HIV fusion inhibitor LP-19

**DOI:** 10.3389/fimmu.2023.1199938

**Published:** 2023-05-15

**Authors:** Lin He, Chen Wang, Yuanyuan Zhang, Huihui Chong, Xiaoyan Hu, Dan Li, Hui Xing, Yuxian He, Yiming Shao, Kunxue Hong, Liying Ma

**Affiliations:** ^1^ State Key Laboratory for Infectious Disease Prevention and Control, National Center for AIDS/STD Control and Prevention, Chinese Center for Disease Control and Prevention, Beijing, China; ^2^ Department of Laboratory Medicine, Yantai Yuhuangding Hospital Affiliated to Qingdao University, Yantai, Shandong, China; ^3^ Beijing Key Laboratory of Emerging Infectious Diseases, Institute of Infectious Diseases, Beijing Ditan Hospital, Capital Medical University, Beijing, China; ^4^ NHC Key Laboratory of Systems Biology of Pathogens, Institute of Pathogen Biology and Center for AIDS Research, Chinese Academy of Medical Sciences and Peking Union Medical College, Beijing, China

**Keywords:** HIV-1, lipopeptide fusion inhibitor, broad-spectrum, antiviral activity, drug resistance barrier

## Abstract

Lipopeptide-19, a HIV fusion inhibitor (LP-19), has showed potent anti-HIV activity. However, there is still limited information of the antiviral activity against different subtype clinical isolates and the drug resistance barrier of LP-19. Therefore, 47 HIV clinical isolates were selected for this study. The viral features were identified, in which 43 strains are CCR5 tropisms, and 4 strains are CCR5/CXCR4 tropisms, and there are 6 subtype B’, 15 CRF01_AE, 14 CRF07_BC, 2 CRF08_BC and 10 URF strains. These 47 viruses were used to detected and analyze the inhibitory activities of LP-19. The results showed that the average 50% inhibitory concentration (IC_50_) and 90% inhibitory concentration (IC_90_) of LP-19 were 0.50 nM and 1.88 nM, respectively. The average IC_50_ of LP-19 to B’, CRF01_AE, CRF07_BC, CRF08_BC, and URF strains was 0.76 nM, 0.29 nM, 0.38 nM, 0.85 nM, and 0.44 nM, respectively. C34 and Enfuvirtide (T-20), two fusion inhibitors, were compared on the corresponding strains simultaneously. The antiviral activity of LP-19 was 16.7-fold and 86-fold higher than that of C34 and T-20. The antiviral activity of LP-19, C34, and T-20 were further detected and showed IC_50_ was 0.15 nM, 1.02 nM, and 66.19 nM, respectively. IC_50_ of LP-19 was about 7-fold and 441-fold higher compared to C34 and T-20 against HIV-1 NL4-3 strains. NL4-3 strains were exposed to increasing concentrations of LP-19 and C34 in MT-2 cell culture. The culture virus was sequenced and analyzed. The results showed that A243V mutation site identified at weeks 28, 32, 38, and 39 of the cell culture in the gp41 CP (cytoplasmic domain) region. NL4-3/A243V viruses containing A243V mutation were constructed. Comparing the antiviral activities of LP-19 against HIV NL4-3 to HIV strains (only 1.3-fold), HIV did not show drug resistance when LP-19 reached 512-fold of the initial concentration under the drug pressure for 39 weeks. This study suggests that LP-19 has broad-spectrum anti-HIV activity, and high drug resistance barrier.

## 1 Introduction

HIV entry inhibitors act in the early stage of HIV infection by preventing the fusion of HIV envelop protein gp41 and co-receptors on target cells, and are considered to have better application prospects in the prevention and treatment of AIDS (1, 2). In the early 1990s, with regard to C-terminal heptad repeat (CHR), it was found that some peptides derived from gp41 could inhibit the fusion of HIV and target cells and resist HIV infection, the most famous of which are T-20 (enfuvirtide) and C34 (3, 4). T-20 became the first to be marketed in 2003 as a HIV entry inhibitor class of antiviral drug (5–9). The mechanism of the two HIV fusion inhibitors is combined with gp41 N-terminal heptad repeat (NHR), thereby preventing CHR on HIV gp41 from combining with NHR to form a six-helix bundle structure. T-20 requires high doses for viral suppression and easily creates drug resistance (10), and a new drug with significantly improved pharmaceutical properties is needed.

He’s team aimed at the conserved pocket site of gp41 (11, 12), and through the integration of multiple design strategies, LP-19 was designed containing the M-T hook structure and the pocket binding sequence, and it is in the state of helix and trimer in solution. LP-19 has showed potent inhibitory effect *in vitro* and in animals. However, HIV is easy to mutate into different subtypes and recombinant viruses (13, 14). Therefore, we tested the inhibitory activities of LP-19 on 47 HIV clinical isolates with different subtypes and recombinants viruses isolated from HIV infected people China.

AIDS patients need to take medicine for life. Drug resistance is a key issue in antiviral therapy. It is reported that there are more and more HIV patients appearing drug resistance to reverse transcriptase inhibitors, protease inhibitors, even T-20 due to long-term treatment. Therefore, this study further tested LP-19 drug resistance, that is the virus was exposed to continuously increasing LP-19 though cell culture passages, and viral mutations were detected to explore the LP-19 resistance barrier.

## 2 Materials and methods

### 2.1 Lipopeptide, C34 and T-20

A lipopeptide-based HIV-1/2 fusion inhibitor, known as LP-19 and formed by adding a fatty acid group (palmitic acid C16) to the C-terminus of 2P23, was designed to target the highly conserved pocket site of gp41with the M-T hook structure. Its sequence is EMTWEEWEKKVEELEKKIEELLK-PEG8-K(C16) (11). The C34 peptide sequence is WMEWDREINNYTSLIHSLIEESQNQQEKNEQELL, and T-20 peptide sequence is YTSLIHSLIEESQNQQEKNEQELLELDKWASLWNWF. The peptides were synthesized by Beijing ZhongkeYaguang Biotechnology Co., Ltd. LP-19, C34, and T-20 were melted at concentrations of 20 μM, 100 μM, and 200 μM and stored in a refrigerator below -20°C for use and diluted when conducting experiments.

### 2.2 Cells, reagents, and viruses

MT-2, HEK 293T, and TZM-bl cell lines and laboratory-adapted HIV strains (NL4-3) were obtained from the National Institutes of Health (NIH) AIDS Research and Reference Reagent Program. MT-2, TZM-bl, and HEK 293T were respectively prepared in RPMI 1640 and DMEM medium, supplemented with 10% fetal bovine serum, 100 ug/mL penicillin and streptomycin. HIV clinical isolates used in the antiviral activities assay were isolated and replicated from HIV infected people in China from the European Research Infrastructures for Poverty Related Diseases project. Informed consent was obtained and signed before sample collection. This study was reviewed by the Institutional Research Ethics Communication of Chinese Center for Disease Control and Prevention (No. X150129355). In the study, 47 culture supernatants of HIV clinical isolates were selected from Anhui, Beijing, Guangxi, and Sichuan respectively. HIV isolates of BJ2015EU14, BJ2015EU16, GX2016EU02, GX2016EU08, XC2014EU09 were national standard strains of pathogenic microorganisms (www.nprc.org.cn).

### 2.3 Viral titration detection

50% cells culture infectious dose (TCID_50_) was expressed as viral titration. HIV titration is determined by setting up serial dilutions of HIV NL4-3 strains or HIV clinical isolates in TZM-bl cell line. One virus of HIV stock was thawed and placed into the first row of the plate in three replicates in 96-well plates, where a 5-fold dilution was firstly made and then continuous dilution for 11 times in triplicate. To each well, 1×10^4^ cells of supplemented DMEM medium were added to 200 μL, and the concentration of DEAE-dextran was 15 μg/mL. And then the plates were incubated at 37°C and 5% CO_2_ for 48 hours. Britelite™ plus Reporter Gene Assay System (PerkinElmer) detected fluorescence, and the viral TCID_50_ was calculated by the Reed-Muench method.

### 2.4 Assay to detect the antiviral activities of HIV fusion inhibitor

HIV NL4-3 strains (200 TCID_50_/well), 1×10^4^ cells (TZM-bl), and LP-19 or C34 or T-20 serial dilutions were added to the 96-well plate, and the final concentration of DEAE-dextran in each well was 15 μg/mL; the experiment was repeated three times. After incubation at 37°C and 5% CO_2_ for 48 hours, the fluorescence value of TZM-bl was detected. The inhibitory percentage calculation is as follows: (average fluorescence value of virus control wells - fluorescence value of LP-19 or C34 or T-20 wells)/(average fluorescence value of virus control wells - average fluorescence value of cell control wells) × 100%. The 50% inhibitory concentration (IC_50_) and 90% inhibitory concentration (IC_90_) of LP-19 lipopeptide or C34 or T-20 was calculated according GraphPad Prism software.

### 2.5 Assay of *in vitro* selection for resistance to LP-19

*In vitro* selection for HIV resistance to LP-19 was performed. 1×10^4^ MT-2 cells were quantitatively infected with NL4-3 strain and inoculated into wells of a 12-well plate. A duplicate well was set up, and positive and negative control wells were set up at the same time (15). 200 TCLD_50_/mL NL4-3 virus was used to infect the MT-2 cells with the addition of 1-fold IC_50_ of LP-19 during cell culture. The cells were incubated at 37°C and 5% CO_2_. The viral replication was monitored by observing the formation of syncytia by optical microscopy. When viruses appeared as a massive syncytium formation, the culture supernatant was harvested at regular time intervals, and the inhibitor concentration was doubled. The culture cells and supernatant were harvested and stored at -80°C. Meanwhile C34 was used as the parallel control of inhibitor.

### 2.6 Amplification and sequencing of HIV *gp41* region

Viral RNA was extracted and purified from infected cells using the QIAamp Viral RNA mini kit (Qiagen). The HIV *gp41* region was amplified using an in-house-designed polymerase chain reaction (PCR) system using first round specific primers (gp41 F1: 5’- AGAGCAGTGGGAATAGGAGCTTTG -3’, gp41 R1: 5’- TGACCACTTGCCACCCATCTTATAGCAA -3’). Second round specific primers (gp41 F2: 5’- TCTTGGGAGCAGCAGGAAGCACTAT -3’; gp41 R2: 5’- GCCCTGTCTTATTCTTCTAGGTATGTGGCG -3’) were used to amplify the HIV-1 *gp41* region (sequenced by TianyiHuiyuan Biotechnology Co., Ltd.). The sequencing results were compared with the pNL4-3 (GenBank: AF324493.2) sequence of NCBI, and the drug resistance-related sites of viral mutations under continuous drug pressure were found.

### 2.7 Site-directed mutagenesis assay

The QuikChange Lightning Site-Directed Mutagenesis Kit (Agilent Technologies) introduces the mutations encoding A243V into the NL4-3 plasmid, and contains the primers and sequences constructed by the mutant site viruses: A243V-F: 5’- GTGAACGGATCCTTGGTACTTATCTGGGACGATC -3’, A243V-R: 5’- GATCGTCCCAGATAAGTACCAAGGATCCGTTCAC -3’. Plasmids containing mutation sites were correctly sequenced (sequenced by TianyiHuiyuan Biotechnology Co., Ltd.).

### 2.8 Transfection

This was carried out based on the instructions of Lipofectamine™ 3000 Reagent (Invitrogen), a cationic lipid-based transfection reagent. 1×10^6^ HEK 293T was inoculated per well in a 6-well plate, and 2 mL DMEM complete medium was added to each well,. The culture was kept overnight in an incubator at a constant temperature of 37°C and 5% CO_2_. When the HEK 293T density reached about 50-70%, the serum-free and antibiotic-free DMEM medium was replaced before the experiment. 125 µL Opti-DMEM and 3.75 µL of Lipofectamine™ 3000 Reagent were added to a 1.5 mL sterilized EP tube and mixed thoroughly. Then 125 µL Opti-DMEM, 5 µL P3000™ Reagent, and 5 µg pNL4-3 were added to another 1.5 mL sterilized EP tube and mixed thoroughly. The mixture in the second tube was added to the first tube, mixed well, and was allowed to stand at room temperature for 15 minutes. The mixture was then evenly and carefully dropped into a six-well plate and incubated at a constant temperature incubator of 37°C and 5% CO_2_ for 4 to 6 hours. Then the complete DMEM medium should be replaced. The supernatants of the virus were harvested 48 hours after transfection, then divided and stored at -80°C in the refrigerator.

## 3 Results

### 3.1 LP-19 is more effective at inhibiting laboratory-adapted HIV-1 strains compared with T-20 and C34

**Figure 1:**
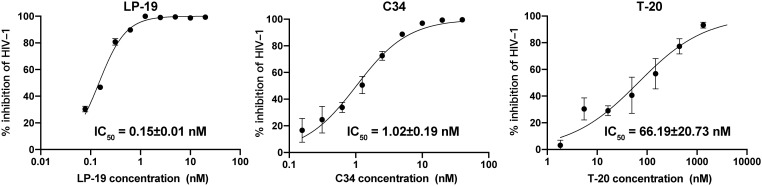
LP-19 exhibits superior viral inhibitory potency compared to C34 and T-20. The IC_50_ data were derived from the results of three independent parallel experiments and expressed as means ± SD.

To evaluate the antiviral activity of LP-19, especially in direct comparison with well-established entry inhibitors T-20 and C34, we utilized TZM-bl reporter assay, whose antiviral effects can be directly measured by the decrease in luciferase signal. The TZM-bl reporter assay revealed that compared with T-20 and C34, LP-19 a 441-fold and 7-fold increased viral inhibitory effect, and the IC_50_ of LP-19, C34, and T-20 against HIV NL4-3 strains was 0.15 ± 0.01 nM, 1.02 ± 0.19 nM, and 66.19 ± 20.73 nM, respectively (**Figure 1**). This result suggested that LP-19 may possess greater antiviral activity than currently available fusion inhibitors.

### 3.2 LP-19 broadly showed antiviral activity against clinical isolates with different subtypes and recombinant isolates

#### 3.2.1 Viral features

Different HIV subtypes may exhibit varying susceptibility to antiretroviral drugs, thus introducing variability in therapeutic outcomes (16, 17). To access the activity of LP-19 across a broad spectrum viruses we used 47 clinical HIV strains isolated from HIV infected people in four provinces in China (**Table 1**). This panel consist of clinical isolates with subtypes B’ (6), CRF_01AE (15), CRF_07BC (14), CRF08_BC (2), and URF (10), which covers circulating strains in China and inter-subtype recombinants. Among the 47 clinical HIV strains, 43 are CCR5-tropic, and 4 are dual tropic (CCR5/CXCR4). Viral P24 antigen levels and viral titration were 5.49 pg/mL and 36,154 TCID_50_/mL respectively (**Table 1**).

**Table 1:** The viral features of 47 clinical isolates and the inhibitory activity of LP-19.

HIV isolate	Viral features				Inhibitory activity of LP-19	
	P24(pg/mL)	Virus subtypes	Tropism*^a^*	TCID_50_/mL*^b^*	LP-19 IC_50_(nM)*^c^*	LP-19 IC_90_(nM)*^c^*
2010096	3.48	B	CCR5	30,000	0.13 ± 0.01	1.22 ± 0.05
2010104	2.68	B	CCR5/CXCR4	50,000	0.77 ± 0.02	5.69 ± 1.36
2010259	8.21	B	CCR5	15,000	0.03 ± 0.00	0.26 ± 0.00
2010968	6.78	B	CCR5	10,000	1.27 ± 0.01	3.23 ± 0.18
BJ2015EU15	7.88	B	CCR5	15,000	2.31 ± 0.05	5.92 ± 1.00
GX2016EU18	0.25	B	CCR5	15,000	0.08 ± 0.00	0.23 ± 0.01
Mean for B’	4.88	B	N/A	22,500	0.76 ± 0.90	2.76 ± 2.60
GX2016EU03	5.38	01_AE	CCR5	50,000	0.96 ± 0.03	3.71 ± 0.38
GX2016EU04	4.41	01_AE	CCR5	50,000	0.74 ± 0.02	2.39 ± 0.12
GX2016EU07	4.40	01_AE	CCR5/CXCR4	50,000	0.44 ± 0.00	1.50 ± 0.05
GX2016EU11	1.53	01_AE	CCR5	30,000	0.88 ± 0.01	2.56 ± 0.18
GX2016EU14	4.19	01_AE	CCR5	10,000	0.01 ± 0.00	0.08 ± 0.00
GX2016EU17	7.16	01_AE	CCR5/CXCR4	30,000	0.02 ± 0.00	0.06 ± 0.01
XC2014EU18	9.89	01_AE	CCR5	30,000	0.06 ± 0.00	0.71 ± 0.01
BJ2015EU01	11.39	01_AE	CCR5	6,250	0.27 ± 0.01	1.03 ± 0.05
BJ2015EU03	5.59	01_AE	CCR5	30,000	0.03 ± 0.00	0.22 ± 0.01
BJ2015EU06	7.94	01_AE	CCR5/CXCR4	30,000	0.20 ± 0.04	0.92 ± 0.04
BJ2015EU09	7.63	01_AE	CCR5	70,000	0.61 ± 0.01	3.18 ± 0.22
BJ2015EU11	8.34	01_AE	CCR5	10,000	0.02 ± 0.00	0.17 ± 0.01
BJ2015EU12	7.12	01_AE	CCR5	30,000	0.10 ± 0.00	0.97 ± 0.06
BJ2015EU14*	6.35	01_AE	CCR5	50,000	0.01 ± 0.00	0.03 ± 0.00
BJ2015EU17	12.40	01_AE	CCR5	15,000	0.01 ± 0.00	0.05 ± 0.01
Mean for 01AE	6.91	01_AE	N/A	32,750	0.29 ± 0.35	1.17 ± 1.23
BJ2015EU02	5.47	07_BC	CCR5	30,000	0.34 ± 0.02	1.83 ± 0.13
BJ2015EU04	5.18	07_BC	CCR5	50,000	0.08 ± 0.00	0.36 ± 0.02
BJ2015EU08	4.52	07_BC	CCR5	70,000	0.51 ± 0.02	3.62 ± 0.23
BJ2015EU13	4.30	07_BC	CCR5	30,000	0.01 ± 0.00	0.05 ± 0.00
GX2016EU01	1.45	07_BC	CCR5	15,000	0.15 ± 0.01	0.99 ± 0.05
GX2016EU05	0.32	07_BC	CCR5	15,000	0.33 ± 0.02	1.53 ± 0.09
GX2016EU08*	4.95	07_BC	CCR5	50,000	0.25 ± 0.02	1.77 ± 0.12
GX2016EU12	5.24	07_BC	CCR5	15,000	0.88 ± 0.02	3.58 ± 0.35
XC2014EU05	7.28	07_BC	CCR5	90,000	0.17 ± 0.01	0.91 ± 0.12
XC2014EU06	2.88	07_BC	CCR5	3,000	0.72 ± 0.12	2.40 ± 0.59
XC2014EU08	5.21	07_BC	CCR5	70,000	0.99 ± 0.02	2.56 ± 0.42
XC2014EU10	1.86	07_BC	CCR5	30,000	0.71 ± 0.00	2.71 ± 0.21
XC2014EU13	11.09	07_BC	CCR5	70,000	0.16 ± 0.01	1.23 ± 0.07
XC2014EU19	8.99	07_BC	CCR5	70,000	0.02 ± 0.00	0.26 ± 0.00
Mean for 07BC	4.91	07_BC	N/A	43,429	0.38 ± 0.33	1.70 ± 1.16
GX2016EU02*	4.46	08_BC	CCR5	50,000	0.78 ± 0.02	1.52 ± 0.20
GX2016EU22	7.26	08_BC	CCR5	30,000	0.10 ± 0.00	1.05 ± 0.10
Mean for 08BC	5.86	08_BC	N/A	40,000	0.85 ± 1.35	2.76 ± 4.28
GX2016EU15	0.24	URF	CCR5	30,000	0.02 ± 0.00	0.17 ± 0.00
GX2016EU09	8.28	URF	CCR5	10,000	0.15 ± 0.00	1.09 ± 0.04
GX2016EU10	4.74	URF	CCR5	50,000	0.16 ± 0.00	0.79 ± 0.02
XC2014EU09*	9.59	URF	CCR5	30,000	4.36 ± 0.04	14.63 ± 1.04
BJ2015EU16*	6.30	URF	CCR5	15,000	0.28 ± 0.01	2.62 ± 0.28
BJ2015EU19	5.52	URF	CCR5	70,000	0.26 ± 0.02	1.01 ± 0.04
GX2016EU13	0.30	URF	CCR5	30,000	0.36 ± 0.01	1.37 ± 0.11
GX2016EU23	0.15	URF	CCR5	50,000	0.77 ± 0.07	1.39 ± 0.28
XC2014EU01	4.32	URF	CCR5	70,000	1.87 ± 0.02	3.52 ± 0.28
XC2014EU20	5.14	URF	CCR5	30,000	0.22 ± 0.03	1.04 ± 0.11
Mean for URF	4.46	URF	N/A	38,500	0.44 ± 0.49	1.29 ± 0.33
Mean	5.49	N/A	N/A	36,154	0.50(0.01~4.36)	1.88(0.03~14.63)

N/A= not applicable. ^a^HIV isolates uses co-receptor CXCR4(CXC chemokine receptor 4), CCR5 (chemokine receptor 5), or both for cells infection. ^b^TCID_50_/mL (50% cells culture infectious dose) is viral titration unit. ^c^IC_50_ or IC_90_ was 50% or 90% inhibitory concentration. The experiment was performed in triplicate, and the data presented are mean values ± standard deviations from an independent experiment. ^*^Viruses were from National Standard Strains of Pathogenic Microorganization (WS/T812-2022) (www.nprc.org.cn).

#### 3.2.2 LP-19 showed high inhibitory activities against 47 clinical isolates

The IC_50_ and IC_90_ of LP-19 on 47 clinical isolated viruses were determined by using gradient dilution. LP-19 exhibited an average IC_50_ of 0.50 nM, ranging from 0.01 nM to 4.36 nM, and IC_90_ was 1.88 nM, ranging from 0.03 nM to 14.63 nM correspondingly (**Table 1**). Further analysis was conducted of LP-19’s inhibitory activities against different HIV subtypes and recombinant viruses. The results showed IC_50_ of LP-19 against subtype B’, CRF01_AE, CRF07_BC, CRF08_BC,and URF were 0.76 nM, 0.29 nM, 0.38 nM, 0.85 nM, and 0.44 nM, respectively. There is no statistically significant difference. The IC_90_ to B’, CRF01_AE, CRF07_BC, CRF08_BC, and URF was 2.76 nM, 1.17 nM, 1.70 nM, 2.76 nM, and 1.29 nM (**Table 1**), respectively. LP-19 effectively inhibits different subtypes with IC_50_ around a few nanomolar concentrations.

#### 3.2.3 Inhibitory effect of LP-19 against 47 HIV isolates compared to C34 and T-20

**Figure 2:**
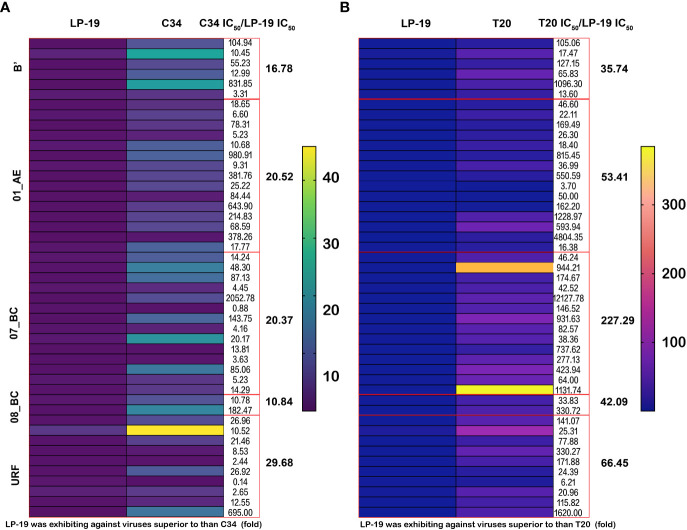
LP-19 is a highly potent inhibitor against 47 clinical HIV isolates viruses compared to C34 and T-20. **(A, B)** The heat map was performed using GraphPad Prism version 8.4.0 for macOS, GraphPad Software San Diego, California USA. The IC_50_ scales are the antiviral activity of LP-19, C34, and T-20 on 47 clinical strains. The yellow-red color in the heat map indicates high IC_50_ and blue-purple color indicates low IC_50_.

A heat map was produced to analyze the inhibitory effect of LP-19 against 47 HIV isolates compared to C34 and T-20. The results showed that average IC_50_ of LP-19, C34, and T-20 was 0.50 nM, 8.35 nM, and 43.00 nM. Inhibitory activities increased 16.7-fold and 86-fold. Compared to C34, IC_50_ of LP-19 for B’, CRF01_AE, CRF07_BC, CRF08_BC, and URF isolates were 16.78-fold, 20.52-fold, 20.37-fold, 10.84-fold, and 29.68-fold respectively. Compared to T-20, the activity of LP-19 to the corresponding subtype strains was 35.74-fold, 53.41-fold, 227.29-fold, 42.09-fold, and 66.45-fold respectively (**Figure 2**).

### 3.3 NL4-3 viruses were not detected with drug mutation under continuously exposed LP-19 for 39 weeks *in vitro*

**Figure 3:**
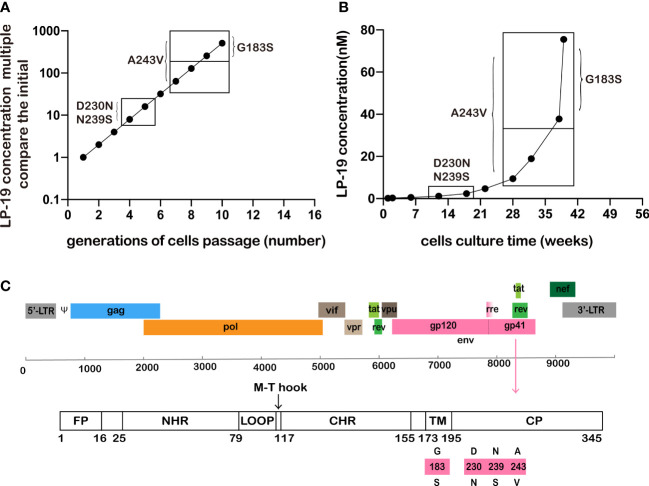
NL4-3 was effectively inhibited at a 512-fold increased concentration of LP-19 over a period of up to 39 weeks. **(A, B)** The HIV strain NL4-3 has a mutation site in the non-NHR region, showing the concentration and time at which the mutation sites appeared. **(C)** Location of HIV gp41 mutation in HXB2. Wild type (amino acids above bars) to mutation type (amino acids below bars), The mutated amino acids site was in HIV gp41 region.

When HIV antiviral drugs are used as a monotherapy, drug-resistant variants inevitably appear. In addition, when a particular HIV drug is used in a multidrug therapy, it is critical to determine the types of drug-resistant mutants that may have evolved or whether these mutations are more resistant or affect inhibitory activity to its antiviral drug (18). Indeed, almost all clinically significant drug resistance mutation arise because of selective drug pressure. Therefore, *in vitro* selection of viruses with resistance to LP-19 was performed (**Figures 3A, B**). HIV NL4-3 virus was cultured in MT-2 cells at an initial concentration of 1-fold IC_50_, when massive syncytia formation was observed, and LP-19 increased as double concentration of IC_50_ in a culture supernatant and cultured continuously for 39 weeks.

When LP-19 concentrations reached 8-fold and 16-fold over the initial concentrations at culture of 12 weeks and 18 weeks, D230N and N239S mutation sites were observed in CP in the gp41 region, but mutation recovery occurred at drug concentrations of 32-fold (22 weeks), 64-fold (28 weeks), 128-fold (32 weeks), 256-fold (38 weeks), and 512-fold (39 weeks). The A243V site was identified at 64-fold, 128-fold, 256-fold and 512-fold drug concentration (cultured for 28, 32, 38, and 39 weeks). The concentration was 256-fold of the initial concentration at 38 weeks, and the G183S site (TM region of gp41) appeared. Until 39 weeks, when the drug concentration reached 512-fold of the initial concentration, no mutations in the NHR gp41 region were found (**Figure 3C**).

**Figure 4:**
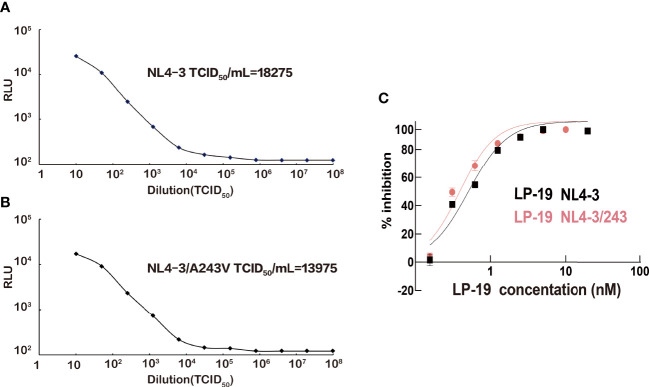
NL4-3 and NL4-3/A243V HIV strains titer and viral sensitivity to LP-19. **(A, B)** Obtain HIV strains NL4-3 and NL4-3/A243V mutation strains in HEK 293T cells. **(C)** Drug inhibition assays were performed in TZM-bl cells to obtain the resistance of the A243V mutated strains of LP-19 compared to the wild strains. RLU, relative light unit.

In order to identify if the A243V mutant site is related to LP-19 resistance, NL4-3/A243V viruses containing the mutant site were produced. The titers of NL4-3 strains and mutant NL4-3/A243V strains were 18,275 TCID_50_/mL and 13,975 TCID_50_/mL, respectively (**Figure 4**). The wild NL4-3 and mutant NL4-3/A243V viruses were detected for viral sensitivity to LP-19. The results showed that LP-19 in the mutant NL4-3/A243V viruses remained at the same level of antiviral activity compared to wild NL4-3 viruses, even is a 1.3-fold activity than before the mutation (**Figure 4C**).

## 4 Discussion

The hydrophobic pocket in the HIV gp41 domain plays an important role in viral fusion and entry into host cells and is an attractive target for the development of HIV fusion/entry inhibitors (3). T-20, a 36-residue natural CHR peptide, is currently the only approved HIV-1 fusion inhibitor by the US FDA for clinical application (6, 19). However, T-20 lacks the N-terminal pocket-binding domain (PBD) critical for high-affinity binding in sequence structure. Its clinical utility is significantly limited by its multiple shortcomings. The hydrophobic pocket of HIV gp41 plays a key role in stabilizing gp41 6-HB core formation and gp41-mediated membrane fusion (20–22). Therefore, the deep pocket of gp41 has been considered as an ideal target site for anti-HIV drugs (23, 24). LP-19 was designed as a short CHR peptide based on the M-T hook structure, which specifically targets the conserved gp41 pocket and avoid the sequence site where T-20 produces drug resistance (25). We tested the inhibitory activity of LP-19 against infection by HIV-1 NL4-3 strains, a HIV laboratory adapted strain. We found that LP-19 was more potent than C34 and T-20, two HIV fusion inhibitors. LP-19 is 7-fold and 441-fold more active than C34 and T-20 against HIV NL4-3 strains. The two residues (Met115 and Thr116) in front of the PBD of CHR peptide adopt a unique M-T hook structure, which can greatly enhance the binding and antiviral activity (26, 27). LP-19 antiviral HIV activity was significantly improved, consistent with some previous studies (19, 25).

Differences in virulence between subtypes and CRFs have been reported (28, 29). Coreceptors used for cell entry have been understood to affect virulence (29). HIV is one of the most genetically diverse pathogens due to its high rate of mutation and recombination. Circulating recombinant forms (CRFs) and unique recombinant forms (URFs) may have a critical impact on drug design (30). Therefore, we focused on the antiviral activity of LP-19 on different subtypes and recombinant viruses with CCR5 (coreceptor) tropisms and CXCR4 (coreceptor) tropisms. LP-19 showed antiviral activity against subtype B’, CRF_01AE, CRF_07BC, CRF08_BC, and URF strains, suggesting broad spectrum, and the activity was significantly improved compared with C34, T-20. The activity of LP-19 to HIV clinical isolates was 16.7-fold and 86-fold higher than that of C34 and T-20. Meanwhile, LP-19 possesses a high antiviral activity HIV clinical isolate with different coreceptor usage (including CCR5 and CXCR4/CCR5). In this study, there are three HIV strains that show lower sensitivity to C34 (1) and T-20 (2). Three viral sequences were detected and found N42S, L54M and A67T mutation (data not be shown) related to C34 and T-20 resistance (31).

HIV develops drug-resistant mutations under treatment pressure, and drug-resistant mutations can be transmitted to treatment-naive individuals, which can lead to rapid virologic failure and potentially limit treatment options. Most clinically significant resistance mutations arise from selective drug pressure. Therefore, we use drug pressure experiments to find drug resistance mutation sites. The effect of drug resistance mutation on virus fitness contributes to understanding the antiretroviral genetic barrier to resistance. By continuously increasing LP-19 *in vitro*, NL4-3 strains were screened for the drug resistance mutation site. NL4-3 strains had no mutation site in the NHR region in the experiment. However, studies on the resistance sites of T-20 report that it has high genetic variability that is increased by the presence of resistance mutations (32). Resistance-associated mutations were initially discovered *in vitro* at position 36-38 of the HR1 domain (33). Primary resistance to T-20 in antiretroviral-native patients have been reported with N42D (34), G36D (34, 35), V38A (35) G36E, N42T, and N43S (36) mutation in the NHR region. The most common substitutions observed in treatment were at positions 36, 38, 40, 42, and 43 (37). The “resistance-associated region” is now spanning positions 32-45 (38, 39). The impact of known mutations on susceptibility to T-20 treatment differs nearly by 100 times (40). When LP-19 concentration was 32-fold over the initial concentration, the A243V site in CP (cytoplasmic domain) appeared. Viruses carrying the A243V substitution remained at a 1.3-fold activity to LP-19. However, we performed *in vitro* selection for HIV resistance to C34. The L44V mutation site began to appear at the 9th generation and until the 12th generation, where the L44V site is located in the gp41 NHR region. The N126K mutation site appeared earlier in the 5th generation until the 12th generation (**Figure S1**). The virus susceptibility of L44V and N126K mutation sites decreased by 6.16-fold and 2.45-fold (**Table S1**). LP-19 exhibits a potentially high resistance barrier compared to C34. It is conceivable that LP-19 primarily targets the highly conserved pocket region of gp41 on the target cell membranes where fusion occurs. LP-19 containing the M-T hook structure provides a highly resistant barrier to the induction of drug resistance (41, 42). Taken together, this shows that LP-19 has highly potent broad-spectrum antiviral activity and a high drug resistance barrier of Lipopeptide HIV fusion inhibitor. Although this study discovered no drug resistance sites, further studies could focus on this. In future studies, peripheral blood mononuclear cells should be infected with viruses and continuously increased with LP-19 for resistant mutation selection.

## Data availability statement

The datasets presented in this study can be found in online repositories. The names of the repository/repositories and accession number(s) can be found below: National Center for Biotechnology Information (NCBI) GenBank, https://www.ncbi.nlm.nih.gov/genbank/, GenBank: AF324493.2.

## Ethics statement

The studies involving human participants were reviewed and approved by Ethics Committee of the National Center for AIDS/STD Control and Prevention, Chinese Centre for Disease Control and Prevention. The patients/participants provided their written informed consent to participate in this study.

## Author contributions

LM, KH, LH, and CW conceived the idea and designed the study. LM and KH supervised the research. LH and CW performed the main experiments and LH wrote the draft manuscript. LH, YZ, and CW were responsible for the sample and information collection. LH collected and analyzed the data. YH and HC provided lipopeptide and revised the manuscript. YS, HX, DL and XH responsible for the information of virus feature and revised the manuscript. All authors contributed to the article and approved the submitted version.

## References

[1] Kaushik-Basu N Basu A Harris D. Peptide inhibition of hiv-1: current status and future potential. BioDrugs (2008) 22(3):161–75. doi: 10.2165/00063030-200822030-00003 18481899

[2] Xiao T Cai Y Chen B. Hiv-1 entry and membrane fusion inhibitors. Viruses (2021) 13(5):735. doi: 10.3390/v13050735 33922579 PMC8146413

[3] Jiang S Lin K Strick N Neurath AR. Hiv-1 inhibition by a peptide. Nature (1993) 365(6442):113. doi: 10.1038/365113a0 8371754

[4] Qadir MI Malik SA. Hiv fusion inhibitors. Rev Med Virol (2010) 20(1):23–33. doi: 10.1002/rmv.631 19827030

[5] Lalezari JP Henry K O’Hearn M Montaner JS Piliero PJ Trottier B. Enfuvirtide, an hiv-1 fusion inhibitor, for drug-resistant hiv infection in north and south america. N Engl J Med (2003) 348(22):2175–85. doi: 10.1056/NEJMoa035026 12637625

[6] Kilby JM Hopkins S Venetta TM DiMassimo B Cloud GA Lee JY. Potent suppression of hiv-1 replication in humans by T-20, a peptide inhibitor of gp41-mediated virus entry. Nat Med (1998) 4(11):1302–7. doi: 10.1038/3293 9809555

[7] Zhang X Ding X Zhu Y Chong H Cui S He J. Structural and functional characterization of hiv-1 cell fusion inhibitor T20. AIDS (2019) 33(1):1–11. doi: 10.1097/QAD.0000000000001979 30096076

[8] Liu S Jiang S. High throughput screening and characterization of hiv-1 entry inhibitors targeting gp41: theories and techniques. Curr Pharm Des (2004) 10(15):1827–43. doi: 10.2174/1381612043384466 15180543

[9] Liu S Jing W Cheung B Lu H Sun J Yan X. Hiv gp41 c-terminal heptad repeat contains multifunctional domains. relation to mechanisms of action of anti-hiv peptides. J Biol Chem (2007) 282(13):9612–20. doi: 10.1074/jbc.M609148200 17276993

[10] Pérez-Alvarez L Carmona R Ocampo A Asorey A Miralles C Pérez de Castro S. Long-term monitoring of genotypic and phenotypic resistance to T20 in treated patients infected with hiv-1. J Med Virol (2006) 78(2):141–7. doi: 10.1002/jmv.20520 16372284

[11] Chong H Xue J Xiong S Cong Z Ding X Zhu Y. A lipopeptide hiv-1/2 fusion inhibitor with highly potent *in vitro*, ex vivo, and *in vivo* antiviral activity. J Virol (2017) 91(11):e00288–17. doi: 10.1128/JVI.00288-17 PMC5432875 28356533

[12] Xiong S Borrego P Ding X Zhu Y Martins A Chong H. A helical short-peptide fusion inhibitor with highly potent activity against human immunodeficiency virus type 1 (HIV-1), HIV-2, and simian immunodeficiency virus. J Virol (2016) 91(1):e01839–16. doi: 10.1128/JVI.01839-16 PMC5165200 27795437

[13] Blassel L Zhukova A Villabona-Arenas CJ Atkins KE Hué S Gascuel O. Drug resistance mutations in hiv: new bioinformatics approaches and challenges. Curr Opin Virol (2021) 51:56–64. doi: 10.1016/j.coviro.2021.09.009 34597873

[14] Nowak M. Hiv mutation rate. Nature (1990) 347(6293):522. doi: 10.1038/347522a0 2215679

[15] Xu W Zhao J Sun J Yin Q Wang Y Jiao Y. The hept analogue wpr-6 is active against a broad spectrum of nonnucleoside reverse transcriptase drug-resistant hiv-1 strains of different serotypes. Antimicrob Agents Chemother (2015) 59(8):4882–8. doi: 10.1128/AAC.00440-15 PMC4505209 26055365

[16] Gartland M Arnoult E Foley BT Lataillade M Ackerman P Llamoso C. Prevalence of gp160 polymorphisms known to be related to decreased susceptibility to temsavir in different subtypes of hiv-1 in the los alamos national laboratory hiv sequence database. J Antimicrob Chemother (2021) 76(11):2958–64. doi: 10.1093/jac/dkab257 PMC8561262 34297843

[17] Li Z Huang Y Ouyang Y Xing H Liao L Jiang S. Mutation covariation of hiv-1 crf07_bc reverse transcriptase during antiretroviral therapy. J Antimicrob Chemother (2013) 68(11):2521–4. doi: 10.1093/jac/dkt228 23788482

[18] Fujiwara T Sato A el-Farrash M Miki S Abe K Isaka Y. S-1153 inhibits replication of known drug-resistant strains of human immunodeficiency virus type 1. Antimicrob Agents Chemother (1998) 42(6):1340–5. doi: 10.1128/AAC PMC105600 9624472

[19] Chong H Wu X Su Y He Y. Development of potent and long-acting hiv-1 fusion inhibitors. AIDS (2016) 30(8):1187–96. doi: 10.1097/QAD.0000000000001073 26919736

[20] Ji H Shu W Burling FT Jiang S Lu M. Inhibition of human immunodeficiency virus type 1 infectivity by the gp41 core: role of a conserved hydrophobic cavity in membrane fusion. J Virol (1999) 73(10):8578–86. doi: 10.1128/JVI.73.10.8578-8586.1999 PMC112878 10482611

[21] Dubay JW Roberts SJ Brody B Hunter E. Mutations in the leucine zipper of the human immunodeficiency virus type 1 transmembrane glycoprotein affect fusion and infectivity. J Virol (1992) 66(8):4748–56. doi: 10.1128/JVI.66.8.4748-4756.1992 PMC241301 1629954

[22] Chen SS Lee CN Lee WR McIntosh K Lee TH. Mutational analysis of the leucine zipper-like motif of the human immunodeficiency virus type 1 envelope transmembrane glycoprotein. J Virol (1993) 67(6):3615–9. doi: 10.1128/JVI.67.6.3615-3619.1993 PMC237711 8497069

[23] Chan DC Kim PS. Hiv entry and its inhibition. Cell (1998) 93(5):681–4. doi: 10.1016/s0092-8674(00)81430-0 9630213

[24] Jiang S Lu H Liu S Zhao Q He Y Debnath AK. N-substituted pyrrole derivatives as novel human immunodeficiency virus type 1 entry inhibitors that interfere with the gp41 six-helix bundle formation and block virus fusion. Antimicrob Agents Chemother (2004) 48(11):4349–59. doi: 10.1128/AAC.48.11.4349-4359.2004 PMC525433 15504864

[25] Chong H Qiu Z Su Y Yang L He Y. Design of a highly potent hiv-1 fusion inhibitor targeting the gp41 pocket. AIDS (2015) 29(1):13–21. doi: 10.1097/QAD.0000000000000498 25562490

[26] Chong H Yao X Qiu Z Qin B Han R Waltersperger S. Discovery of critical residues for viral entry and inhibition through structural insight of hiv-1 fusion inhibitor cp621-652. J Biol Chem (2012) 287(24):20281–9. doi: 10.1074/jbc.M112.354126 PMC3370210 22511760

[27] Chong H Yao X Sun J Qiu Z Zhang M Waltersperger S. The m-T hook structure is critical for design of hiv-1 fusion inhibitors. J Biol Chem (2012) 287(41):34558–68. doi: 10.1074/jbc.M112.390393 PMC3464562 22879603

[28] Taylor BS Sobieszczyk ME McCutchan FE Hammer SM. The challenge of hiv-1 subtype diversity. N Engl J Med (2008) 358(15):1590–602. doi: 10.1056/NEJMra0706737 PMC2614444 18403767

[29] Asjö B Morfeldt-Månson L Albert J Biberfeld G Karlsson A Lidman K. Replicative capacity of human immunodeficiency virus from patients with varying severity of hiv infection. Lancet (1986) 2(8508):660–2. doi: 10.1016/S0140-6736(86)90169-8 2429124

[30] Streeck H Li B Poon AF Schneidewind A Gladden AD Power KA. Immune-driven recombination and loss of control after hiv superinfection. J Exp Med (2008) 205(8):1789–96. doi: 10.1084/jem.20080281 PMC2525594 18625749

[31] Araújo LA Junqueira DM de Medeiros RM Matte MC Almeida SE. Naturally occurring resistance mutations to hiv-1 entry inhibitors in subtypes b, c, and crf31_bc. J Clin Virol (2012) 54(1):6–10. doi: 10.1016/j.jcv.2012.01.005 22336085

[32] Si-Mohamed A Piketty C Tisserand P LeGoff J Weiss L Charpentier C. Increased polymorphism in the hr-1 gp41 env gene encoding the enfuvirtide (T-20) target in hiv-1 variants harboring multiple antiretroviral drug resistance mutations in the pol gene. J Acquir Immune Defic Syndr (2007) 44(1):1–5. doi: 10.1097/01.qai.0000243118.59906.f4 17075396

[33] Rimsky LT Shugars DC Matthews TJ. Determinants of human immunodeficiency virus type 1 resistance to gp41-derived inhibitory peptides. J Virol (1998) 72(2):986–93. doi: 10.1128/JVI.72.2.986-993.1998 PMC124569 9444991

[34] Peuchant O Capdepont S Ragnaud JM Aurillac-Lavignolle V Thiébaut R Fleury H. Primary resistance to enfuvirtide (T20) in recently HIV-1 infected, antiretroviral-naive patients from the ANRS aquitaine cohort. Antivir Ther (2007) 12(4):559–62. doi: 10.1177/135965350701200413 17668565

[35] Wei X Decker JM Liu H Zhang Z Arani RB Kilby JM. Emergence of resistant human immunodeficiency virus type 1 in patients receiving fusion inhibitor (T-20) monotherapy. Antimicrob Agents Chemother (2002) 46(6):1896–905. doi: 10.1128/AAC.46.6.1896-1905.2002 PMC127242 12019106

[36] Cardoso LP Stefani MM. High level of multidrug resistance mutations in HIV type 1 pol gene and resistance-associated mutations to enfuvirtide (T-20) among antiretroviral-experienced patients from central Brazil. AIDS Res Hum Retroviruses (2009) 25(10):943–50. doi: 10.1089/aid.2009.0060 19792869

[37] Sista PR Melby T Davison D Jin L Mosier S Mink M. Characterization of determinants of genotypic and phenotypic resistance to enfuvirtide in baseline and on-treatment HIV-1 isolates. AIDS (2004) 18(13):1787–94. doi: 10.1097/00002030-200409030-00007 15316339

[38] Greenberg ML Cammack N. Resistance to enfuvirtide, the first HIV fusion inhibitor. J Antimicrob Chemother J Antimicrob Chemother (2004) 54(2):333–40. doi: 10.1093/jac/dkh330 15231762

[39] Zöllner B Feucht HH Schröter M Schäfer P Plettenberg A Stoehr A. Primary genotypic resistance of HIV-1 to the fusion inhibitor T-20 in long-term infected patients. AIDS (2001) 15(7):935–6. doi: 10.1097/00002030-200105040-00015 11399967

[40] Lu J Sista P Giguel F Greenberg M Kuritzkes DR. Relative replicative fitness of human immunodeficiency virus type 1 mutants resistant to enfuvirtide (T-20). J Virol (2004) 78(9):4628–37. doi: 10.1128/jvi.78.9.4628-4637.2004 PMC387671 15078945

[41] Chong H Qiu Z Su Y He Y. The n-terminal T-T motif of a third-generation hiv-1 fusion inhibitor is not required for binding affinity and antiviral activity. J Med Chem (2015) 58(16):6378–88. doi: 10.1021/acs.jmedchem.5b00109 26256053

[42] Chong H Yao X Qiu Z Sun J Qiao Y Zhang M. The m-T hook structure increases the potency of hiv-1 fusion inhibitor sifuvirtide and overcomes drug resistance. J Antimicrob Chemother (2014) 69(10):2759–69. doi: 10.1093/jac/dku183 24908047

