# Supplementary material for: Broad-spectrum anti-HIV activity and high drug resistance barrier of lipopeptide HIV fusion inhibitor LP-19
Source: Front Immunol. 2023 May 15;14:1199938. doi: 10.3389/fimmu.2023.1199938 (PMC10225588; doi:10.3389/fimmu.2023.1199938)
Supplement: Supplementary file 1 [file DataSheet_1.docx]

Supplementary Material

Broad-spectrum anti-HIV activity and high drug resistance barrier of Lipopeptide HIV fusion inhibitor LP-19

Lin He^†^, Chen Wang^†^, Yuanyuan Zhang, Huihui Chong, Xiaoyan Hu, Dan Li, Hui Xing, Yuxian He, Yiming Shao, Kunxue Hong^*^ and Liying Ma^*^

*** Correspondence:** Liying Ma: mal@chinaaids.cn; Kunxue Hong: hongkx@chinaaids.cn

# Supplementary Figures and Tables


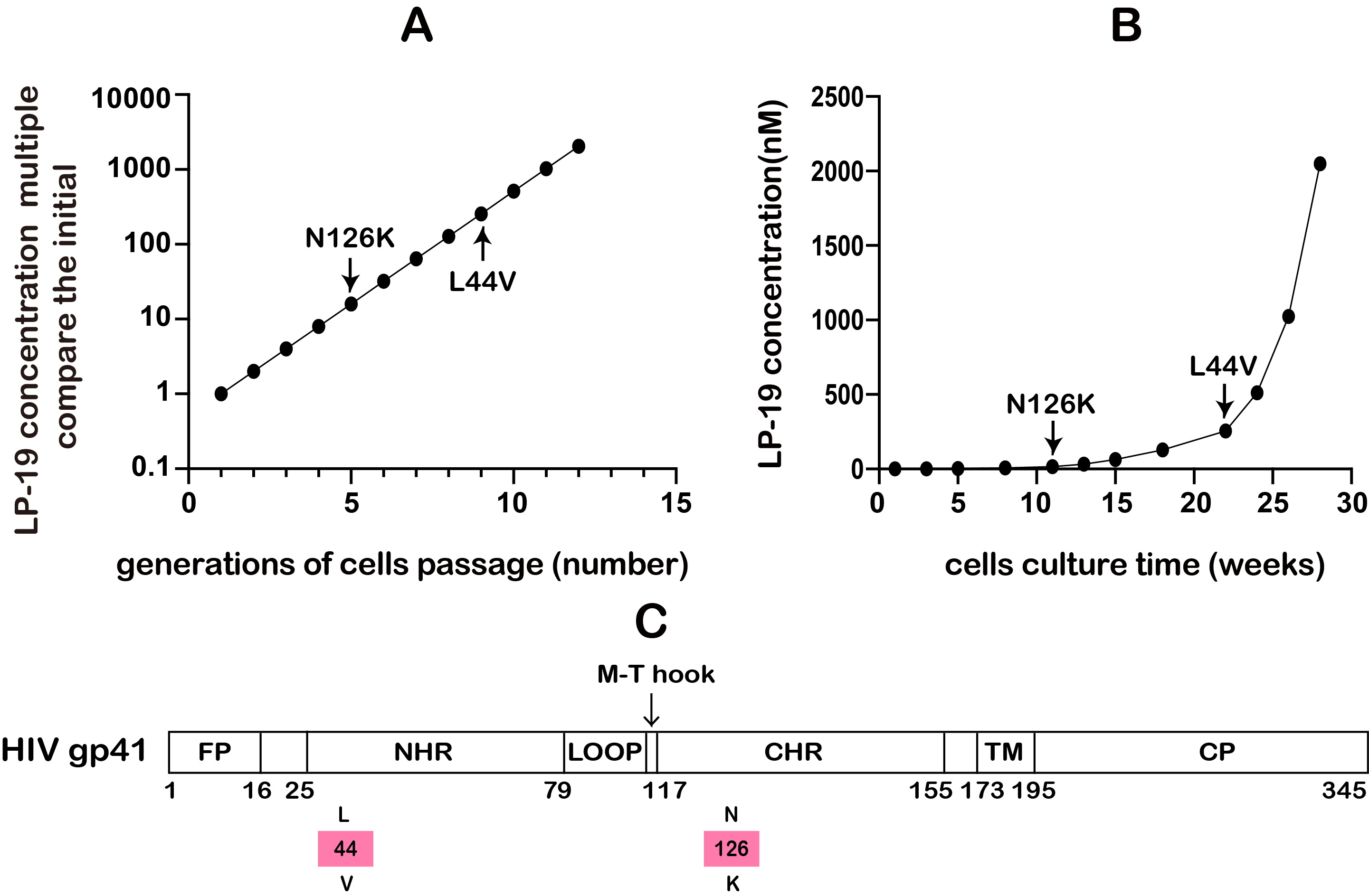


**Fig.S1** Two drug resistance site were obtained by *in vitro* C34 drug resistance screening. (A, B) Generations number of cells passage, C34 drug concentration, and culture time when gp41 mutations site appeared *in vitro*. The N126K mutation site was detected from the 5^th^ to the 12^th^ generation. The L44V mutation site were detected from the 9^th^ to the 12^th^ generation. (C) Location of HIV gp41 mutation in HXB2. Wild type (amino acids above bars) to mutation type (amino acids below bars), The mutated amino acids site was in HIV gp41 region.

**Table S1**. TCID_50_ of NL4-3 viruses containing the L44V and N126K mutant sites and analysis of its sensitivity to C34 HIV-1 fusion inhibitor

| **Mutant strains** | **TCID_50_/mL** | **C34** | |
| --- | --- | --- | --- |
|  |  | **IC_50_ (nM)** | **n-fold** |
| NL4-3 | 69877 | 0.98±0.03 | 1.00 |
| NL4-3/L44V | 13975 | 6.04±1.66 | 6.16 |
| NL4-3/N126V | 18275 | 2.40±0.52 | 2.45 |
